# N-Acetylcysteine Attenuates the Increasing Severity of Distant Organ Liver Dysfunction after Acute Kidney Injury in Rats Exposed to Bisphenol A

**DOI:** 10.3390/antiox8100497

**Published:** 2019-10-21

**Authors:** Wachirasek Peerapanyasut, Anongporn Kobroob, Siripong Palee, Nipon Chattipakorn, Orawan Wongmekiat

**Affiliations:** 1 Renal Physiology Unit, Department of Physiology, Faculty of Medicine, Chiang Mai University, Chiang Mai 50200, Thailand; wachirasek_pee@cmu.ac.th; 2 Division of Physiology, School of Medical Sciences, University of Phayao, Phayao 56000, Thailand; anongporn.ko@up.ac.th; 3 Cardiac Electrophysiology Research and Training Center, Department of Physiology, Faculty of Medicine, Chiang Mai University, Chiang Mai 50200, Thailand; siripong.pal@cmu.ac.th (S.P.); nipon.chat@cmu.ac.th (N.C.)

**Keywords:** acute kidney injury, bisphenol A, ischemia and reperfusion, liver, mitochondria, N-acetylcysteine, oxidative stress, remote organ injury

## Abstract

Distant organ liver damage after acute kidney injury (AKI) remains a serious clinical setting with high mortality. This undesirable outcome may be due to some hidden factors that can intensify the consequences of AKI. Exposure to bisphenol A (BPA), a universal chemical used in plastics industry, is currently unavoidable and can be harmful to the liver. This study explored whether BPA exposure could be a causative factor that increase severity of remote liver injury after AKI and examined the preventive benefit by N-acetylcysteine (NAC) in this complex condition. Male Wistar rats were given vehicle, BPA, or BPA + NAC for 5 weeks then underwent 45 min renal ischemia followed by 24 h reperfusion (RIR), a group of vehicle-sham-control was also included. RIR not only induced AKI but produced liver injury, triggered systemic oxidative stress as well as inflammation, which increasing severity upon exposure to BPA. Given NAC to BPA-exposed rats diminished the added-on effects of BPA on liver functional impairment, oxidative stress, inflammation, and apoptosis caused by AKI. NAC also mitigated the abnormalities in mitochondrial functions, dynamics, mitophagy, and ultrastructure of the liver by improving the mitochondrial homeostasis regulatory signaling AMPK-PGC-1α-SIRT3. The study demonstrates that NAC is an effective adjunct for preserving mitochondrial homeostasis and reducing remote effects of AKI in environments where BPA exposure is vulnerable.

## 1. Introduction

Acute kidney injury (AKI) is a major health problem worldwide which occurs in various clinical settings. Despite remarkable advances in preventive and therapeutic strategies, AKI remains associated with an unacceptably high mortality rate [1,2]. The impact of AKI on other organs injury, also called ‘remote or distant organ injury’, is accepted as a major cause of this undesirable outcome [2,3,4]. Notably, the remote effect of AKI on the liver is an important point of concern as convincing evidence exist that AKI patients with liver injury exhibit poor prognosis and increased mortality [5]. Also, there may be other hidden factors that can have an influence on remote liver injury by AKI. This issue requires further investigation.

Nowadays, repeated exposure to certain chemicals is common in our daily lives and becoming a global public health concern. As the liver is an important organ for detoxification, this situation can lead to deterioration of liver function which may intensify the remote effects of AKI and worsen its outcomes. Bisphenol A (BPA) is one of the important chemicals produced in the largest volume worldwide as a precursor of polycarbonate plastics and epoxy resins [6]. Despite the long-standing question about its safety, BPA continues to be used in a variety of consumer products, particularly food packaging and medical devices, as it is difficult to develop a chemical replacement which is more economical and safe [7]. Epidemiological data reveal that more than 90% of general populations had detectable level of BPA [8], while those of the occupational exposure had higher BPA level than general individuals about 70 times [9]. These data indicate that humans are vulnerable to BPA exposure and this condition may be unavoidable. Most importantly, subclinical liver impairment after short-term BPA exposure has recently been revealed [10].

On the basis that humans are constantly exposed to BPA, while the incidence of AKI is high and unpredictable, the simultaneous occurrence of both events is indeed feasible. In this case, it will be a very serious and uncontrollable situation that can lead to increased morbidity and mortality in AKI patients especially in those with latent or subclinical liver impairment. Thus, strategy to protect or minimize the dysfunction of the liver as a remote organ under this complex condition is a challenge to improve clinical outcome during AKI.

N-acetylcysteine (NAC) is a well-known drug that is widely used clinically with a high safety report. It also has several pleiotropic effects such as antioxidant, anti-inflammation and anti-apoptosis [11]. Studies have successfully reported the benefits of NAC in preventing AKI under various conditions, including renal ischemia-reperfusion [12] and chemical-induced nephrotoxicity [13,14]. In addition, the role of NAC as a mitochondrial protective agent for organ preservation has recently been highlighted [13,15]. Since mitochondrial alterations and oxidative stress are closely related to the adverse consequences of AKI as well as BPA toxicity [16,17], NAC may be a useful candidate to cope with remote liver injury caused by AKI under this vulnerable circumstance of BPA exposure.

In the present study, we first tested whether exposure to BPA prior to AKI incidence could aggravate the remote effect of AKI on the liver. Then, the potential of NAC to defend against any adverse impact of BPA in this complex condition of AKI was assessed. The investigation also extended to evaluate whether the adenosine monophosphate-activated protein kinase, peroxisome proliferator-activated receptor gamma coactivator 1-alpha, sirtuin 3 (AMPK-PGC-1α-SIRT3) axis, a crucial signaling mechanism for regulating mitochondrial homeostasis and redox balance, was related to the therapeutic possibility of NAC.

## 2. Materials and Methods

### 2.1. Animal Preparation

Male Wistar rats (200–250 g) were obtained from the National Laboratory Animal Center Mahidol University, Salaya Thailand. Rats were housed in an animal holding room under standard conditions (temperature 24 ± 1 °C, 55 ± 5% relative humidity, regular 12 h light/dark cycle). Animals received food and water ad libitum and allowed to acclimatize for 1 week prior to the experiment. All study protocols were approved by the Institutional Animal Care and Use Committee of the Faculty of Medicine, Chiang Mai University (project number 08/2561).

### 2.2. Induction of Acute Kidney Injury

The renal ischemia-reperfusion (RIR) model was chosen as the representative of AKI because it is the most common cause of AKI and occurs in high incidence. RIR was induced under aseptic conditions using the surgical procedure previously described [17]. Briefly, rats were anesthetized by intraperitoneal injection of 37.5 mg/kg zoletil (Virbac Laboratories, Carros, France) mixed with 2.25 mg/kg xylazine (Bayer Animal Health, Leverkusen, Germany). Both renal pedicles were exposed through midline incision and occluded using non-traumatic vessel clips for 45 min followed by 24 h reperfusion. Upon reperfusion, the clips were removed, and the kidneys were monitored for color change to confirm blood reflow before closing the incision. Similar surgical procedures except clamping of the renal pedicles were applied to the sham-operated group. All animals were closely observed until full recovery from anesthesia before being returned to their cages.

### 2.3. Experimental Designs

The first experiment was carried out as a preliminary study to verify the occurrence of AKI as well as remote organ liver injury and to assess how BPA exposure affected this condition. Twenty-four male Wistar rats were equally divided into 2 sets. Each set consisted of 3 groups (*n* = 4 each) which was treated orally with vehicle (corn oil) or BPA (Sigma Chemical Co., St. Louis, MO, USA) at the doses of 5 and 50 mg/kg, respectively. A 5 mg/kg BPA was used since it is a no-observed-adverse-effect level (NOAEL) in rat [18], while a 50 mg/kg selection was based on previous studies regarding BPA adverse effect [19,20]. After 5 weeks of treatment, rats in the first set underwent sham operation and those in the second set were subjected to RIR induction. Blood samples were taken at the end of reperfusion to determine renal and liver functions, including systemic oxidative stress and inflammatory levels.

The second experiment was undertaken to further explore the possible benefits and mechanisms involved in the protection by NAC against remote effects of AKI on the liver under a complex circumstance of AKI combined with BPA pre-exposure. Since the severity of AKI-induced remote liver injury was increasing when BPA concentration increased (preliminary data obtained from the first experiment), we chose to assess the efficacy of NAC at BPA 50 mg/kg. In this experiment, four groups of male Wistar rats (*n* = 6 each) were studied. Group I (VS) and Group II (VIR) received vehicle (corn oil) via oral gavage for 5 weeks then subjected to sham operation and RIR induction, respectively. Group III (BIR) and Group IV (BNIR) were given BPA 50 mg/kg for 5 weeks prior to RIR induction. Apart from BPA, rats in Group IV (BNIR) were also treated orally with NAC 100 mg/kg given 60 min before BPA administration. The selected dose and treatment regimen for NAC was based on previous reports in rats showing its potential to protect against cognitive dysfunction induced by BPA [21] as well as hepatotoxicity induced by acetaminophen [22].

Blood samples were collected after 24 h reperfusion, the animals were then sacrificed by intraperitoneal injection with an overdose of pentobarbital sodium. The liver was immediately removed for mitochondrial study, and light and electron microscopic examinations, while the remainders were snap-frozen in liquid nitrogen and stored at −80 °C for further analysis.

### 2.4. Assessments of Renal and Liver Functions

Serum levels of urea nitrogen (BUN), creatinine (SCr), aspartate aminotransferase (AST), and alanine aminotransferase (ALT) were measured using AU480 chemistry analyzer (Beckman Coulter, Inc., Brea, CA, USA).

### 2.5. Assessments of Systemic as well as Liver Oxidative Stress and Inflammation

Systemic and liver oxidative stress were assessed by determinations of nitric oxide (NO), malondialdehyde (MDA), and reduced glutathione (GSH) levels in serum and liver samples, respectively, using commercial kits (BioAssay Systems, Hayward, CA, USA). A pro-inflammatory cytokine, tumor necrosis factor-alpha (TNF-α), was also detected in both serum and liver samples using ELISA kit (Invitrogen, Thermo Fisher Scientific, Waltham, MA, USA).

### 2.6. Light Microscopic Studies

Liver tissues were fixed in 10% neutral buffered formaldehyde and subsequently dehydrated in ascending grades of alcohol, cleared in xylene, and embedded in paraffin wax. Paraffin sections (4 μm) were cut and stained with hematoxylin and eosin (H&E). The sections were examined under light microscope by a pathologist blinded to the treatment protocol.

### 2.7. Electron Microscopic Studies

Transmission electron microscopy was used to examine the liver ultrastructure. A slight modification of previously published protocol [17] was applied. Liver tissues were fixed with 2.5% glutaraldehyde in 0.1 M phosphate buffer (pH 7.4) overnight at 4 °C then subsequently post-fixed in 2% phosphate-buffered osmium tetroxide. After dehydration in graded ethanol, the tissues were washed in propylene oxide, and embedded in Epon resin using EMbed-812 embedding kit (Electron Microscopic Sciences, Hatfield, PA, USA). The obtained ultra-thin sections (60–80 nm thick) were mounted on copper grids, stained with uranyl acetate and lead citrate, and inspected with JEM-2200 FS transmission electron microscope (JEOL, Tokyo, Japan).

### 2.8. Preparations of Liver Mitochondrial Fractions and Proteins

The liver mitochondrial fraction was prepared according to method described by Sayeed et al. [23]. Briefly, liver tissue was homogenized in ice-cold lysis buffer containing 230 mM mannitol, 70 mM sucrose, 1 mM ethylene diamine tetra acetic acid (EDTA), and 10 mM Tris-HCl, pH 7.4. Differential centrifugation technique was used for isolation of mitochondrial fraction. The final mitochondrial pellets were resuspended in ice-cold respiration buffer (250 mM sucrose, 5 mM KH_2_PO_4_, 10 mM Tris-HCl, 2 mg/mL bovine serum albumin (BSA), pH 7.2) and mitochondrial protein concentration was determined by bicinchoninic acid (BCA) assay using bovine serum albumin as standard [24].

### 2.9. Determination of Liver Mitochondrial Reactive Oxygen Species (ROS)

Liver mitochondrial ROS was measured using non-fluorescent 2′,7′-dichlorofluorescein diacetate (DCFDA) (Sigma Chemical Co., St. Louis, MO, USA). Liver mitochondria were incubated with 2 μM DCFDA at 25 °C for 60 min [25]. DCFDA can diffuse through the mitochondrial membrane and is converted to a highly fluorescent dichlorofluorescein (DCF) by ROS in the mitochondria. The fluorescent intensity was measured with excitation wavelength at 485 nm and emission wavelength at 530 nm using Synergy^TM^ H4 fluorescene microplate reader (BIOTEK^®^ Instruments, Inc., Winooski, VT, USA). The elevation of the fluorescent intensity indicated the increase in mitochondrial ROS production. The ROS levels were expressed as arbitrary units of fluorescence intensity of DCF.

### 2.10. Determination of Liver Mitochondrial Membrane Potential Change (ΔΨm)

JC-1 (5,5′,6,6′-tetrachloro-1,1′,3,3′-tetraethylbenzimi-dazocarbocyanine iodide) dye (Sigma Chemical Co., St. Louis, MO, USA) was used to evaluate a change in mitochondrial membrane potential. The dye accumulates and spontaneously forms aggregate with intense red color in high ΔΨm, whereas it remains in the monomeric form that yields green fluorescence with low ΔΨm. Mitochondrial depolarization was determined by a reduction in the ratio of aggregate to monomer form. Isolated liver mitochondria were incubated with JC-1 dye for 30 min at 37 °C [25]. The red fluorescence was excited at a wavelength of 535 nm and the emission detected at 590 nm while the green fluorescence was excited at a wavelength of 485 nm and the emission detected at 530 nm using a fluorescent microplate reader (BIOTEK^®^ Instruments, Inc., Winooski, VT, USA).

### 2.11. Determination of Liver Mitochondrial Swelling

Light-scattering technique was used for determination of mitochondrial swelling [26]. Briefly, the change in absorbance of liver mitochondrial suspension at 540 nm was measured for 15 min using a microplate reader (Synergy^TM^ H4, BIOTEK^®^ Instruments, Inc., Winooski, VT, USA). The swelling of mitochondria was represented by a decrease in the absorbance of the mitochondrial suspension.

### 2.12. Western Blot Analysis

Liver protein was extracted by homogenization of the frozen liver tissues in a pre-cold lysis buffer with 1% protease inhibitor. Bio-Rad protein assay kit (Bio-Rad Laboratories, Hercules, CA, USA) was used for total protein quantification. The proteins were then loaded onto 10% SDS-polyacrylamide gel electrophoresis (SDS-PAGE), and transferred to nitrocellulose membranes. The membranes were blocked for 1 h with 5% bovine serum albumin in Tris-buffered saline-Tween 20 (TBST) and subsequently probed with primary antibodies against Ac-SOD2, Bax (Abcam, Cambridge, MA, USA), p-AMPK^Thr172^, PGC-1α (Millipore Corporation, Billerica, MA, USA), Bcl-2, pro-caspase3, cleaved-caspase3, AMPK, SIRT3, dynamin 1-like protein (DRP1), p-DRP1^Ser616^, mitofusin 2 (MFN2), superoxide dismutase 2 (SOD2), PTEN-induced putative kinase 1 (PINK1), PARKIN, voltage-dependent anion channels (VDAC) (Cell Signaling Technology, Danvers, MA, USA) and a loading control β-actin (Santa Cruz Biotechnology, Santa Cruz, CA, USA) overnight at 4 °C. After washed with TBST, bound antibody was detected using a horseradish peroxidase-conjugated secondary antibody and visualized with an enhanced chemiluminescence (ECL) detection reagent and exposed using ChemiDoc™ Touch Imaging System (Life Science AP, Bio-Rad, Hercules, CA, USA). The signal intensities were quantified using Image J program (National Institute of Health, Bethesda, MD, USA).

### 2.13. Statistical Analysis

All data are expressed as means ± SEM and analyzed using Graphpad Prism 5 software (GraphPad Software, San Diego, CA, USA). Comparison among the groups was performed using one-way analysis of variance (ANOVA) followed by Fisher’s Least-Significant Difference post-hoc test. Statistical significance was considered at a *p*-value less than 0.05.

## 3. Results

### 3.1. RIR Induces Remote Organ Liver Injury and the Severity is Increasing upon BPA Exposure

As shown in Table 1, all animals that experience RIR, whether being exposed to BPA or not, exhibited the rise in serum AST and ALT, in addition to the accumulation of blood urea nitrogen and serum creatinine when compared to their related sham-controls (*p* < 0.05). Significant increases in circulating levels of NO, MDA, and TNF-α but decrease in GSH were also evident in animals encountered RIR. However, all the changes after RIR incidence were significantly intensified when BPA exposure was pre-existing, and the severity increased upon increasing BPA concentration.

This preliminary outcome provided evidence to support that RIR not only induced AKI but produced systemic oxidative stress, inflammation, and remote liver injury. The results also demonstrated that pre-exposure to BPA has an adverse impact on remote effect of AKI on the liver. In the next step, we aimed at exploring whether NAC could be advantage to protect or reduce the injury of liver against this complicated disorder. BPA at 50 mg/kg was chosen for challenging the effectiveness of NAC since this dose caused liver impairment even upon sham operation. Moreover, we expect that if NAC could prevent the serious injury induced by AKI under this high concentration of BPA; it would definitely have a potency to protect the liver against any adverse consequences caused by AKI or BPA alone.

### 3.2. NAC Therapy Attenuates the Increased Severity of Remote Liver Injury after RIR under BPA-Exposed Condition

**Figure 1:**
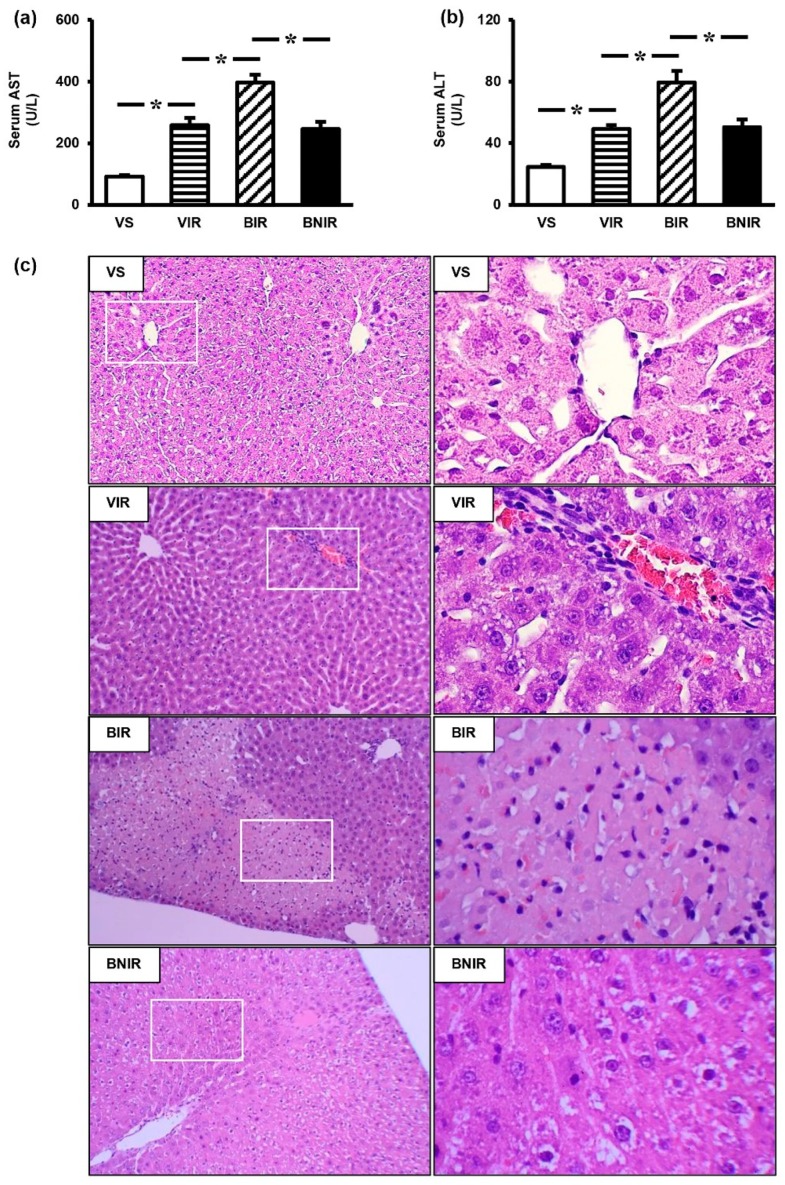
Effects of N-acetylcysteine (NAC) on liver function (**a,b**) and histopathological alterations (**c**): H&E 10× left column and 40× right column) after renal ischemia-reperfusion (RIR) in bisphenol A (BPA)-exposed rats. Values are means ± SEM (*n* = 6). VS: vehicle-treated sham group; VIR: vehicle-treated RIR group; BIR: BPA (50 mg/kg)-treated RIR group; BNIR: BPA (50 mg/kg) plus NAC (100 mg/kg)-treated RIR group. * *p* < 0.05 between groups.

Consistent with our preliminary study, RIR induced liver dysfunction as indicated by markedly increased serum AST (Figure 1a) and ALT (Figure 1b) in comparison with the sham controls (*p* < 0.05), and the severity was augmented (*p* < 0.05) in BPA-exposed rats. Most importantly, the deterioration of liver function resulting from AKI coexisted with BPA exposure was significantly reduced when pretreatment with NAC. Histological studies provided further evidence to support the protection by NAC in this circumstance (Figure 1c). Liver tissues obtained from the vehicle-treated sham group (VS) showed normal histological findings. In the vehicle-treated RIR group (VIR), abnormal characteristics—such as dilated sinusoid, centrilobular congestion, and leukocyte (mainly lymphocyte) infiltration in portal tracts—were observed. Apart from these findings, focal hepatocellular necrosis was also detected in the BPA-treated RIR group (BIR), where all these histological alterations were diminished in the BPA plus NAC-treated RIR group (BNIR).

### 3.3. NAC Reduces Oxidative Stress and Inflammation in the Systemic Circulation as well as Remote Liver Organ after RIR under BPA-Exposed Condition

**Figure 2:**
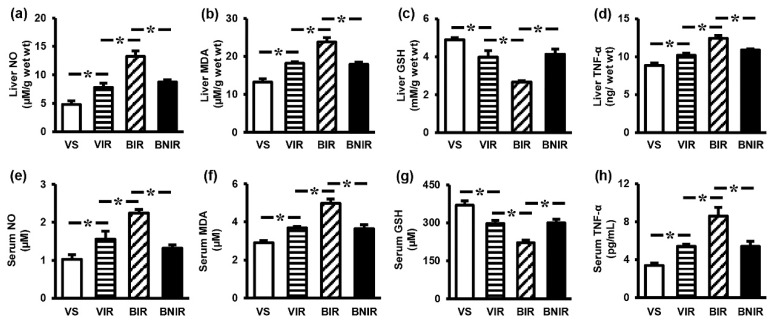
Effects of N-acetylcysteine (NAC) on oxidative stress and inflammatory markers in liver tissue (**a–d**), and serum (**e–h**) after renal ischemia-reperfusion (RIR) in bisphenol A (BPA) exposed rats. Values are means ± SEM (*n* = 6). VS: vehicle-treated sham group; VIR: vehicle-treated RIR group; BIR: BPA (50 mg/kg)-treated RIR group; BNIR: BPA (50 mg/kg) plus NAC (100 mg/kg)-treated RIR group. NO: nitric oxide; MDA: malondialdehyde; GSH: reduced glutathione; TNF-α: tumor necrosis factor-α. * *p* < 0.05 between groups.

To investigate whether the alleviation of liver injury by NAC was due to its ability to reduce oxidative injury as well as inflammatory response, the liver tissue and serum levels of NO, MDA, GSH, and TNF-α were measured. Significant increases in both the liver tissue and serum levels of NO (Figure 2a,e), MDA (Figure 2b,f), and TNF-α (Figure 2d,h) along with decreases in GSH (Figure 2c,g) were shown in animals encountered RIR incidence. The severity of these changes was exacerbated in BPA-treated RIR rats (*p* < 0.05). Supplementation with NAC to rats pre-exposure to BPA significantly attenuated systemic including remote liver oxidative stress and inflammation caused by RIR.

### 3.4. Maintenance of Remote Liver Mitochondrial Function Contributes to the Protection by NAC after RIR under BPA-Exposed Condition

**Figure 3:**
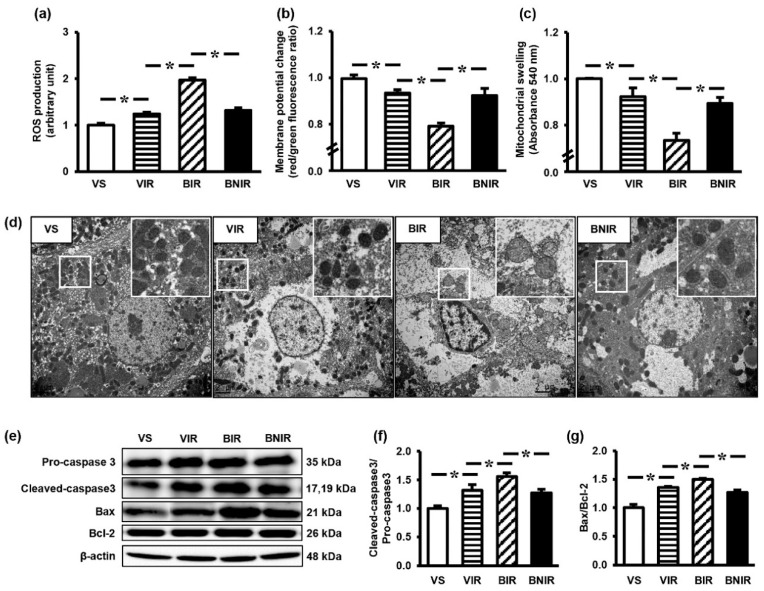
Mitochondrial function (**a–c**), transmission electron micrographs (**d**), representative images of western blots (**e**), the quantitative analyses of cleaved-caspase3/pro-caspase3 (**f**), and Bax/Bcl-2 (**g**) in the liver following renal ischemia-reperfusion (RIR) in rats with bisphenol A (BPA) exposure and N-acetylcysteine (NAC) treatment. Values are means ± SEM (*n* = 6). VS: vehicle-treated sham group; VIR: vehicle-treated RIR group; BIR: BPA (50 mg/kg)-treated RIR group; BNIR: BPA (50 mg/kg) plus NAC (100 mg/kg)-treated RIR group. * *p* < 0.05 between groups. Transmission electron micrographs were at original magnification (2000×) and the boxed areas are magnified in the right upper panel.

To assess whether the beneficial role of NAC was associated with the protection of mitochondrial integrity, we therefore evaluated the function, morphology, and proteins involved in apoptotic process of the mitochondria. Ischemia followed by reperfusion of the kidneys led to liver mitochondrial dysfunction as indicated by a significant increase in mitochondrial ROS production (Figure 3a), a significant decrease in mitochondrial membrane potential change (Figure 3b), and a significant swelling of the mitochondria (Figure 3c). The intensity of RIR-induced mitochondrial dysfunction was significantly elevated in case of BPA pre-exposure, but this increment was significantly mitigated by NAC therapy.

Electron microscopic examination of the liver was conducted to attest the remote consequence of RIR together with BPA exposure at ultrastructural level (Figure 3d). Liver tissues from vehicle-treated sham rats (VS) showed normal appearance of hepatocytes and mitochondria as characterized by normal hepatic intercellular conjunction with numerous, dense, and uniform mitochondria. Induction of RIR in the vehicle-treated rats (VIR) produced mildly enlargement of hepatocyte and slightly reduction in the number of mitochondria. However, substantial alterations were evident in the rats that exposure to BPA prior to RIR induction (BIR) as noticed by incompact hepatic intercellular conjunction, severe mitochondrial swelling with loss of cristae and massive decrease in mitochondrial quantity. These ultrastructural alterations were reduced considerably in the group received NAC treatment (BNIR).

In line with the changes in liver mitochondrial function and morphology, the expressions of mitochondrial apoptotic proteins cleaved-caspase3/pro-caspase3 (Figure 3e,f) and Bax/Bcl-2 (Figure 3e,g) were also significantly increased in the liver following RIR. The expression levels of these proteins were further increased (*p* < 0.05) by exposure to BPA before RIR induction, while pretreatment with NAC significantly blunted these modifications.

### 3.5. Signaling Transmission Through AMPK-PGC-1α-SIRT3 is Involved in the Remote Liver Mitochondrial Protection by NAC after RIR under BPA-Exposed Condition

**Figure 4:**
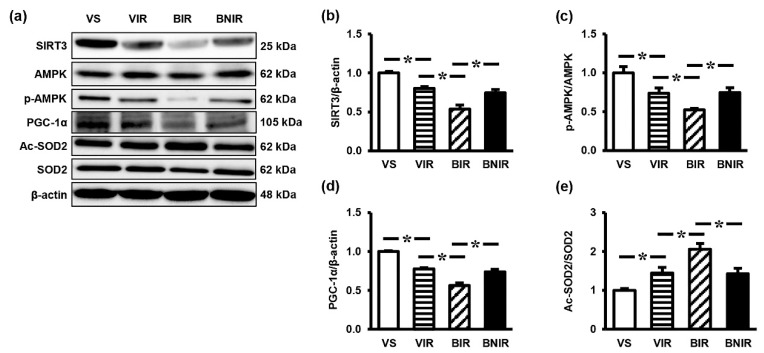
Representative images of western blots (**a**) and the quantitative analyses of SIRT3 (**b**), p-AMPK/AMPK (**c**), PGC-1α (**d**), and Ac-SOD2/SOD2 (**e**) in the liver following renal ischemia-reperfusion (RIR) in rats with bisphenol A (BPA) exposure and N-acetylcysteine (NAC) treatment. Values are means ± SEM (*n* = 6). VS: vehicle-treated sham group; VIR: vehicle-treated RIR group; BIR: BPA (50 mg/kg)-treated RIR group; BNIR: BPA (50 mg/kg) plus NAC (100 mg/kg)-treated RIR group. * *p* < 0.05 between groups.

To explore whether the enhancement of mitochondrial homeostasis underlay the mitochondrial protection by NAC in these settings, the liver expressions of regulatory proteins p-AMPK, AMPK, PGC-1α, SIRT3, Ac-SOD2, and SOD2 were investigated. RIR significantly decreased the protein levels of SIRT3 (Figure 4a,b), p-AMPK/AMPK ratio (Figure 4a,c) and PGC-1α (Figure 4a,d) but increased Ac-SOD2/SOD2 ratio (Figure 4a,e). The degree of alterations was significantly greater in the BPA-treated RIR group (BIR) compared to the vehicle-treated RIR group (VIR). As expected, treatment with NAC significantly attenuated these changes.

**Figure 5:**
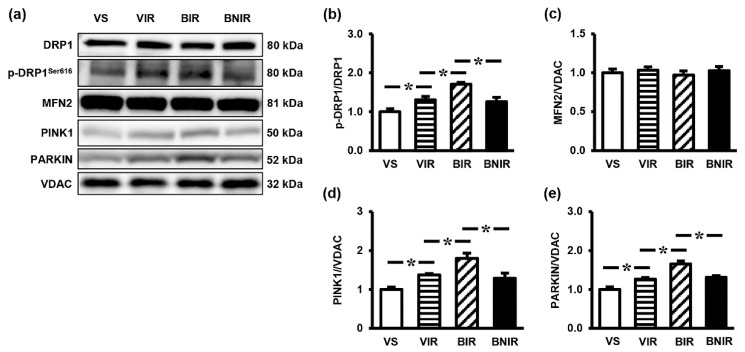
Representative images of western blots (**a**) and the quantitative analyses of p-DRP1/DRP1 (**b**), MFN2 (**c**), PINK1 (**d**), and PARKIN (**e**) in the liver following renal ischemia-reperfusion (RIR) in rats with bisphenol A (BPA) exposure and N-acetylcysteine (NAC) treatment. Values are means ± SEM (*n* = 6). VS: vehicle-treated sham group; VIR: vehicle-treated RIR group; BIR: BPA (50 mg/kg)-treated RIR group; BNIR: BPA (50 mg/kg) plus NAC (100 mg/kg)-treated RIR group. * *p* < 0.05 between groups.

Mitochondrial dynamic and mitophagy are the key processes in mitochondrial homeostasis, which are regulated through AMPK-PGC-1α-SIRT3 axis. Accordingly, the expressions of proteins involved in mitochondrial dynamic (p-DRP1, DRP1, MFN2) and mitophagy (PINK1, PARKIN) were determined. Liver tissues from the vehicle-treated animals that underwent RIR induction showed significant increases in the protein levels of p-DRP1/DRP1 (Figure 5a,b), PINK1 (Figure 5a,d) and PARKIN (Figure 5a,e), with no significant changes in MFN2 (Figure 5a,c). Similar patterns of protein expressions were also found in the BPA-treated RIR rats, but to a greater extent (*p* < 0.05). The observed overexpression of proteins in this complicated event was significantly decreased when treated with NAC.

## 4. Discussion

The novel and significant finding from this study is the benefit of NAC to prevent the effect of BPA in increasing the severity of remote liver injury caused by renal ischemia and reperfusion. Studies also provide evidence to suggest that NAC diminishes this complex disorder by preserving mitochondrial homeostasis, improving mitochondrial function, and reducing oxidative injury within the remote organ. Activation of SIRT3, at least in part, through AMPK-PGC-1α-SIRT3 axis, is involved in the therapeutic outcomes of NAC in this condition.

Distant organ injury is a serious complication that result in poor prognosis and high mortality after acute kidney injury. Among several remote organ consequences, hepatic dysfunction is accepted as the leading cause of this undesirable outcome. In the present study, remote liver injury is clearly observed after ischemia and reperfusion of the kidneys, as evidenced by increased liver enzymes and abnormal liver histology in combination with the existence of oxidative stress and inflammation both in the systemic circulation and liver tissues. These characteristics are consistent with previous studies indicating the occurrence of distant organ dysfunction following acute kidney injury [4,27,28]. Interestingly, our results also demonstrated that the injury of remote liver is aggravated by BPA exposure, while supplementation with NAC is effectively impeded this adverse impact of BPA. Evidence obtained also indicated that the beneficial outcome of NAC is associated with the reduction of mitochondrial damage as supported by decreased mitochondrial ROS production, inhibited mitochondrial membrane potential (ΔΨm) dissipation, reduced mitochondrial swelling, diminished mitochondrial apoptosis and minimized mitochondrial structural changes. This is in line with previous studies exhibited the mitochondrial protection by NAC in other disease models [15,29,30]. At present, however, the molecular mechanisms including signaling pathway that contribute to the protection of mitochondria by NAC remain largely unidentified.

Mitochondria are highly dynamic organelles that not only produce cellular energy but also modulate several cellular functions. The precise regulation of mitochondrial quality (morphology and function) as well as quantity by mitochondrial dynamics (fission-fusion), mitophagy and mitochondrial biogenesis is a key aspect to maintain mitochondrial homeostasis and, thus, normal cell and organ functions [31]. Derangement of these machineries lead to the development and progression of diseases related to mitochondrial dysfunction [32]. In recent years, regulation of mitochondrial homeostasis has been emphasized not only as an underlying cause of disease but also as a therapeutic strategy in many pathological settings [33,34]. Since mitochondria are the important targets of ischemia-reperfusion injury including BPA adverse effect, it is possible that the benefits of NAC observed in our study may be due to its potential to retain the homeostatic mechanisms within the mitochondria. To address this prospect, we further examined the major proteins involved in mitochondrial fission (dynamin 1-like protein: DRP1), fusion (mitofusin 2: MFN2), mitophagy (PTEN-induced putative kinase 1: PINK1 and PARKIN), and biogenesis (peroxisome proliferator-activated receptor gamma coactivator 1-alpha: PGC-1α).

Our results showed significant increases in the expressions of p-DRP1/DRP1, but no changes in MFN2, in the liver following RIR induction. Similar patterns but higher levels of expressions were observed in rats exposed to BPA prior to RIR induction. The findings point toward a shift of mitochondrial fission-fusion balance to fission, causing mitochondrial fragmentation, morphological alterations, and subsequent functional impairments. This is in lines with previous studies showing overexpression of DRP1 in several experimental models including renal ischemia-reperfusion [35,36] and BPA-induced neurotoxicity [37]. Normally, the dysfunctional mitochondria are appropriately removed by the process of mitophagy [31]. However, our study demonstrated the superfluous increases in PINK1/PARKIN protein expression following RIR, with the highest levels detected in the BPA-exposed RIR group. These findings clearly indicate a disruption in the mitophagy balance. Indeed, recent evidence suggests that excessive mitophagy plays a pivotal role both in ischemia-reperfusion injury [38] and the deleterious outcomes following BPA exposure [39]. Apart from mitophagy, mitochondrial biogenesis is also an integral aspect of the maintenance of mitochondrial quality [31,32,40]. The tight regulation of these opposing processes is essential for mitochondrial turnover and maintain homeostasis in response to intracellular stress and mitochondrial malfunction [40]. Although mitochondrial biogenesis is achieved by the activation of several transcription factors, PGC-1α is accepted as a central regulator because it serves as a transcriptional co-activator and orchestrates the activity of diverse transcription factors involved in mitochondrial proliferation [40]). In the present study, PGC-1α expression was suppressed upon RIR induction in the BPA pre-exposed rats, indicating a disturbance in mitochondrial biogenesis. Consistent with the present data, downregulation of PGC-1α has previously been reported in experimental models of ischemia-reperfusion injury [41] and BPA toxicity [10]. In addition, activation of PGC-1α has been shown to trigger mitochondrial proliferation, improve mitochondrial respiration, and alleviate mitochondrial defects in several models of mitochondrial diseases [42]. Above all, our results showed that supplementation of NAC to the BPA-exposed rats successfully blocked the modifications of p-DRP1/DRP1, PINK1/PARKIN, and PGC-1α in the remote liver organ after RIR induction. These findings strongly indicate that the protection by NAC to this complex condition is mediated via its ability to maintain the homeostatic machineries within the mitochondria.

Because PGC-1α is the central controller of the mitochondrial quality control, the signaling pathways that eventually converge on the PGC-1α also affect the mitochondrial homeostasis [31,40]. Accumulating evidence show that phosphorylation by AMP-activated protein kinase (AMPK) and deacetylation by mitochondrial silent information regulator 3 (SIRT3) are required for PGC-1α activation [32,43]. Accordingly, the AMPK-PGC-1α-SIRT3 axis is suggested as a crucial signaling pathway involved in the regulation of mitochondrial homeostasis and mitochondrial redox balance [34,44]. Interestingly, we also noticed significant increases in the expressions of p-AMPK/AMPK and SIRT3 in conjunction with PGC-1α in the BPA-exposed RIR group treated with NAC. Previous studies in various organs and under several pathological settings also demonstrated the reduction in mitochondrial injury upon increases in the levels of AMPK, PGC-1α, and SIRT3 [44,45]. Thus, it is proposed that NAC exerts mitochondrial protection in our study model, in part, through the activation of AMPK-PGC-1α-SIRT3 signaling pathway. However, the in-depth mechanisms by which NAC regulates this axis require further investigation.

Oxidative stress plays a crucial role in the pathogenesis of ischemia-reperfusion injury [17] as well as BPA adverse effect [16]. Our study also demonstrated the existence of systemic oxidative stress originated from the injured kidneys following ischemia and reperfusion. This situation could affect the liver and result in remote liver injury. According to the well recognize antioxidant abilities of NAC, reducing free radical production as well as boosting antioxidant defense system is suggested as other mechanism for the protection of remote liver injury by NAC in the present study. This suggestion is confirmed by our findings of decreases in nitric oxide (NO) and malondialdehyde (MDA) in association with increases in antioxidant glutathione (GSH) levels both in the systemic circulation and in the liver tissues upon NAC treatment. We also found the reduction of ROS production and the increased expression of acetylated-superoxide dismutase 2/superoxide dismutase 2 (Ac-SOD2/SOD2) within the liver mitochondria after NAC treatment. SOD2 is a key enzyme responsible for scavenging superoxide anion and is also a substrate of SIRT3 within the mitochondrial matrix. The physical binding of SIRT3 and SOD2 results in the deacetylation and activation of SOD2, thereby inhibiting oxidative damage and maintaining mitochondrial redox balance [46]. In view of the major source of ROS generation, mitochondrial ROS must be tightly controlled as it plays an important role in the regulation of cellular signaling and dictates biological outcome [47]. Based on our findings, it is most likely that the upregulation of SIRT3 after NAC supplementation is a crucial mechanism responsible for mitochondrial protection against remote liver injury following RIR under the vulnerable condition of BPA exposure. Studies in various experimental models also show that upregulation of SIRT3 reduces mitochondrial injury and preserves organ function, while deletion of SIRT3 exacerbates the injury [33,34,44,48].

Another interesting point of this study is that we first, to our knowledge, proved the importance of SIRT3 in the remote liver organ injury following AKI. Our study demonstrated that mitochondrial damage, together with oxidative injury, was noticed in the liver after RIR induction and these alterations were related to the reduction of SIRT3 proteins expression. Thus, activation of SIRT3 may become an alternative therapeutic target for preventing extra-organ injury caused by AKI in the future.

One last point that should be mentioned is that, despite remarkable impacts of BPA and therapeutic benefits of NAC, our results are not able to clearly indicate where BPA and NAC are really acting. BPA as well as NAC may exert its direct impact on the liver after AKI. Alternatively, NAC may do nothing to the underlying liver damage caused by remote effect of AKI, but only counteract the effects induced by BPA exposure. Future works on this aspect are warrant.

## 5. Conclusions

This study indicates that repeated exposure to BPA have an adverse consequence on AKI-induced remote organ injury in the liver, which could contribute to the high mortality rate of this serious clinical setting. Oxidative stress-mediated mitochondrial disorders via AMPK-PGC-1α-SIRT3 axis appear to be the underlying cause of this deleterious outcome. The present findings raise awareness of the harmful effect of BPA exposure, particularly in those with high risk to AKI. The evidence obtained also demonstrates that supplementation with NAC is effective at protecting against the dramatic impact of this important industrial chemical.

## Figures and Tables

**Table 1:** Effects of renal ischemia and reperfusion (RIR) on kidney and liver functions, serum oxidative stress, and inflammatory markers in bisphenol A (BPA)-exposed rats.

Parameters		Sham				RIR	
	Veh	BPA5	BPA50		Veh	BPA5	BPA50
BUN (mg/dL)	21.95 ± 0.81 ^a^	22.53 ± 0.92 ^a^	22.88 ± 0.83 ^a^		67.33 ± 4.38 ^b^	90.11 ± 0.92 ^c^	105.52 ± 5.65 ^d^
SCr (mg/dL)	0.29 ± 0.00 ^a^	0.30 ± 0.01 ^a^	0.29 ± 0.01 ^a^		1.36 ± 0.14 ^b^	2.15 ± 0.11 ^c^	3.06 ± 0.20 ^d^
AST (U/L)	80.25 ± 2.75 ^a^	78.50 ± 1.94 ^a^	115.00 ± 3.49 ^b^		233.50 ± 26.93 ^c^	311.50 ± 15.19 ^d^	384.25 ± 19.75 ^e^
ALT (U/L)	23.75 ± 1.31 ^a^	24.25 ± 1.89 ^a^	31.75 ± 1.31 ^b^		45.50 ± 1.55 ^c^	56.50 ± 3.59 ^d^	70.75 ± 3.61 ^e^
NO (µM)	0.93 ± 0.13 ^a^	0.94 ± 0.11 ^a^	0.97 ± 0.10 ^a^		1.32 ± 0.11 ^b^	1.87 ± 0.08 ^c^	2.35 ± 0.10 ^d^
MDA (µM)	2.95 ± 0.12 ^a^	3.06 ± 0.12 ^a^	3.01 ± 0.21 ^a^		3.63 ± 0.04 ^b^	4.40 ± 0.35 ^c^	5.50 ± 0.17 ^d^
GSH (µM)	363.50 ± 11.09 ^a^	365.00 ± 12.15 ^a^	356.33 ± 14.70 ^a^		294.50 ± 10.22 ^b^	250.00 ± 3.74 ^c^	210.33 ± 11.26 ^d^
TNF-α (pg/mL)	3.24 ± 0.34 ^a^	3.09 ± 0.40 ^a^	3.31 ± 0.47 ^a^		5.52 ± 0.34 ^b^	7.51 ± 0.27 ^c^	9.98 ± 0.42 ^d^

Values are means ± SEM (*n* = 4). BPA 5, 50: BPA-treated group at 5 and 50 mg/kg, respectively; BUN: blood urea nitrogen; SCr: serum creatinine; AST: aspartate aminotransferase; ALT: alanine aminotransferase; NO: nitric oxide; MDA: malondialdehyde; GSH: reduced glutathione; TNF-α: tumor necrosis factor-alpha. ^a–e^ Mean values with different letters in the same row are significantly different (*p* < 0.05).
